# A novel and highly sensitive nanocatalytic surface plasmon resonance-scattering analytical platform for detection of trace Pb ions

**DOI:** 10.1038/srep24150

**Published:** 2016-04-13

**Authors:** Lingling Ye, Guiqing Wen, Huixiang Ouyang, Qingye Liu, Aihui Liang, Zhiliang Jiang

**Affiliations:** 1 Key Laboratory of Ecology of Rare and Endangered Species and Environmental Protection of Ministry Education, Guangxi Normal University, Guilin 541004, China

## Abstract

Gold nanoparticles (AuNP) have catalysis on the reaction of HAuCl_4_-H_2_O_2_. The produced AuNP have strong resonance Rayleigh scattering (RRS) effect and surface-enhanced resonance Raman scattering (SERS) effect when Victoria blue B (VBB) and rhodamine S (RhS) were used as probes. The increased RRS/SERS intensity respond linearly with the concentration of gold nanoparticles (AuNP_B_) which synthesized by NaBH_4_ over 0.038–76 ng/mL, 19–285 ng/mL, 3.8–456 ng/mL respectively. Four kinds of tested nanoparticles have catalysis on the HAuCl_4_-H_2_O_2_ particles reaction. Thus, a novel nanocatalysis surface plasmon resonance-scattering (SPR-S) analytical platform was developed for AuNP. The DNAzyme strand hybridized with the substrate strand to form double-stranded DNA (dsDNA) which couldn’t protect AuNP_c_ to aggregate to AuNP_c_ aggregations, having strong RRS effect. Upon addition of Pb^2+^, dsDNA could be cracked by Pb^2+^ to produce single-stranded DNA (ssDNA) that adsorbed on the AuNPc surface to form AuNPc-ssDNA conjugates. The conjugates have strong catalysis on HAuCl_4_-H_2_O_2_ reaction. With increased Pb^2+^ concentration, the concentration of AuNPc-ssDNA increased and lead to the catalytic activity stronger. The increased RRS intensity responds linearly with Pb^2+^ concentration over 16.7–666.7 nmol/L. The SERS intensity responded linearly with the concentration of Pb^2+^ over 50–500 nmol/L.

Nanoparticles are of advantages of unique optical, electrical and chemical properties, and have been used for protein and nucleic acid analysis, biosensors, biochips and nanocatalytic analysis^1^,^2^,^3^,^4^,^5^,^6^,^7^,^8^. Noble metal nanoparticles have high electron density, good biocompatibility, good catalysis and good stability, easy preparation, so it has attracted people’s attentions. Haruta found that nanogold was a good catalyst which load on the transition metal oxides^9^, not only has high catalytic activity for CO oxidation at low temperature, but also have the advantages of good water resistance, stability and the enhanced effect of humidity^10^. It has broken the traditional ideas that nanogold has no catalytic activity. In the analysis of trace contaminants, nanocatalysis provides opportunities to establish a high sensitive and selective analysis method to amplify analytical signal, and improve selectivity that combine with immunoreaction and nucleic acid aptamer reaction^11^,^12^,^13^,^14^,^15^,^16^. Xu *et al*.^15^ have reported a new light scattering method for determination of nucleic acid using immunonanogold catalytic amplification, with a detection limit of 10 fmol/L. Our group developed two new technologies including immunonanocatalyis and aptamer-modified nanoparticle catalysis, that have been used for detecting 7.2 pg/mL urine albumin and 0.09 ng/mL IgG^14^,^17^,^18^. These demonstrate that exploring new highly sensitive nanocatalysis analytical reaction is very significant. Among the nanoparticles, nanogold in solution has best stability and strong catalysis. H_2_O_2_ not only has no effect for subsequent SPR research but also is colorless, accessible and with non-toxic product. As far as we know, there are no reports about H_2_O_2_-HAuCl_4_-nanogold catalytic analytical reaction and used for the SPR-S analysis platform.

The SPR-S techniques included the RRS and SERS, which the former is elastic and the later is inelastic scattering that both were based on the nanoparticle scattering. RRS is simple, sensitive spectral analysis method and has been used for protein, nucleic acid and metal ions analysis^19^,^20^,^21^,^22^,^23^. Lead is a harmful heavy metal, which has been listed as key detection project in food, drugs, environmental pollutants and supervision inspection. Based on the reaction of Pb^2+^ reacting with excessive I^-^ to form [PbI_4_]^2−^, and further associated with rhodamine 6 G (Rh6G) to produce particles with a strong RRS peak, Luo *et al*.^24^ developed a RRS method for detection of Pb^2+^ as low as 0.04 μg/mL. Luo *et al*.^25^ reported a RRS method for detection Pb^2+^ as low as 1.0 nmol/L, based on the binding of Pb(II) with thrombin and aptamer. Pb^4+^ was reduced to PbH_4_ gas by NaBH_4_ and the gas trapped by Au^3+^ to form nanogold that exhibited a RRS effect at 286 nm. This principle was used to detect Pb^2+^ as low as 7.0 × 10^−8 ^mol/L^26^. Based on the dsDNA cracked by Pb(II) to release a short single-stranded DNA that conjugated gold nanoparticles (AuNPs) to form a stable AuNPs-ssDNA complex, and its nanocatalysis of HAuCl_4_-vitamin C particle reaction, a sensitive RRS method was developed for detection of Pb(II)^27^. However, there are no reports about the HAuCl_4_-H_2_O_2_ nanogold catalysis SPR-RS analytical platform being utilized to detect trace Pb^2+^, combing with the DNA enzymes cracked reaction. SERS is a sensitive and selective molecular spectrometry, based on the molecular probes adsorbed on rough surface of nanoparticles^28^,^29^,^30^,^31^,^32^,^33^. Although there are many SERS detection techniques, a few SERS quantitative methods have been reported, with good accuracy, simplicity and practicality. Liu *et al*.^34^ proposed a SERS biosensor to detect lead ion, combining the DNAzyme cracking and nanocatalytic reaction. Zhang *et al*.^35^ used the prepared tree-shape nanogold-DNA as a signal amplifier to fabricate a SERS biosensor for detection of 100 pmol/L Pb^2+^. A label-free rhodamine 6G SERS probe was reported for detection of trace Pb(II) in Au_core_Ag_shell_ nanosol substrate, based on the Pb(II) cracking the DNAmyze^36^. However, there are no reports about aptamer combining with the nanocatalysis of H_2_O_2_-HAuCl_4_ in SERS quantitative analysis of Pb^2+^. In this paper, we have considered the new nanocatalytic reaction of AuNP-HAuCl_4_-H_2_O_2_, and two new SPR methods were developed for detection of Pb(II), combining the analysis platform with the DNAzyme cracking.

## Results

### RRS spectra

**Figure 1:**
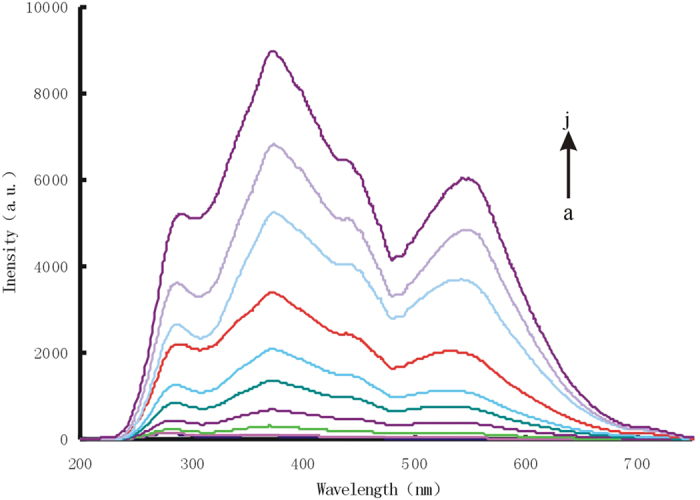
RRS spectrum of AuNP_c_-HAuCl_4_-H_2_O_2_ system. (**a**) 4.48 μmol/L HAuCl_4_+0.67 mmol/L HCl+3.33 mmol/L H_2_O_2_ (**b**) a+1.9 ng/mL AuNP_c_; (**c**) a+3.8 ng/mL AuNP_c_; (**d**) a+7.6 ng/mL AuNP_c_; (**e**) a+19 ng/mL AuNP_c_; (**f**) a+34.2 ng/mL AuNP_c_; (**g**) a+47.ng/mL AuNP_c_; (**h**) a+85.5 ng/mL AuNP_c_; (**i**) a+133 ng/mL AuNP_c_; (**j**) a+161.5 ng/mL AuNP_c_.

The RRS signals of small particle size gold, silver, platinum and palladium nanosol are very weak. Different concentrations of AgNO_3_ were added to preparation of AuNR_1_, AuNR_2_ and AuNR_3_, with the diameter of 32 nm, 37 nm and 43 nm respectively that RRS values gradually reduced (Fig. S1). With the increase of AuNR concentration, the RRS peak linear increased at 370 nm (Fig. S1D). Nanoparticles can catalytic hydrogen peroxide reduction of HAuCl_4_ under the condition of 0.67 mmol/L HCl, and with the increase of nanogold solution concentration, the RRS intensity of system linear increase at 370 nm (Fig. 1, Fig. S2). The catalytic activity of AuNP_B_ was better than that of AuNP_c_ because the particle size of AuNP_B_ was smaller than AuNPc, which lead to the surface energy more larger and the surface of AuNP_B_ nanoparticles can absorb more HAuCl_4_. Different particle size of AuNR was added as catalyst, with the AuNR concentration increased , the RRS peak linear increased at 370 nm (Figs S3–S5). When AgNPs, PdNPs, PtNPs nanosol solution was used as catalyst, with the increase of nanoparticles concentration, the RRS peak linear increased at 370 nm (Fig. S6). It can be used to quantitative of HAuCl_4_ and H_2_O_2_ through this catalytic system, with the increase of HAuCl_4_ concentration, the RRS peak of system linear increased and color from colorless gradually became red (Fig. S7), with the increase of H_2_O_2_ concentration, the RRS peak of system linear increased (Fig. S8). When AuNPc-ssDNA solution was used as catalyst, AuNP_c_ modified by aptamer catalytic activity is stronger than AuNP_c_ solution, with the increase of AuNPc-ssDNA concentration, the RRS peak linear increased at 370 nm (Fig. S9).

**Figure 2:**
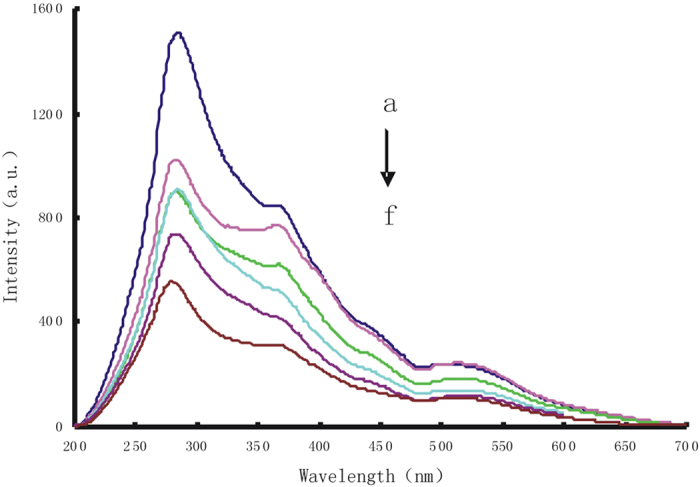
RRS spectrum of Pb^2+^ -aptamer-AuNP_c_ system. (**a**) 4 mM pH 8.0 Tris-HCl-50 μL hybrid solution-9.55 μg/mLAuNP_C_-10 mmol/LNaCl; (**b**) a+0.125 μmol/L Pb^2+^; (**c**) a+0.175 μmol/L Pb^2+^; (**d**) a+0.25 μmol/L Pb^2+^; (**e**) a+0.375 μmol/L Pb^2+^; (**f**) a+0.425 μmol/L Pb^2+^.

**Figure 3:**
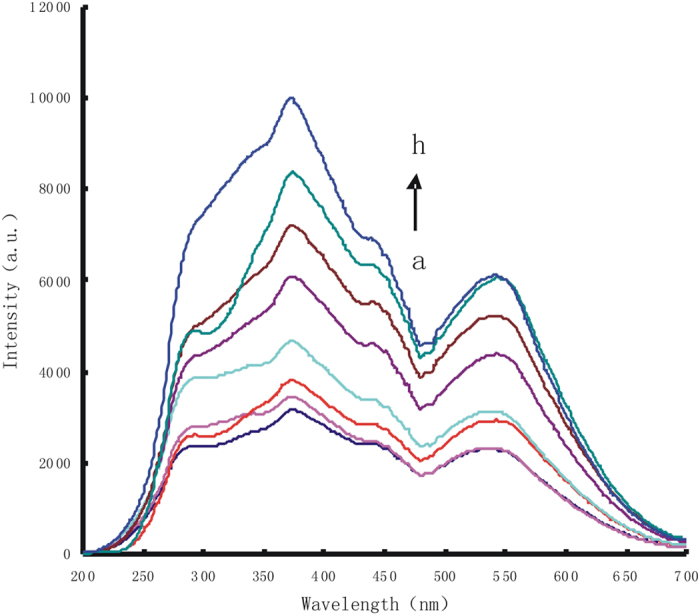
RRS spectrum of Pb^2+^ reaction solution-HAuCl_4_-H_2_O_2_ nanocatalytic system. (**a**) 4.48 μmol/L HAuCl_4_+0.67 mmol/L HCl+3.33 mmol/L H_2_O_2_; (**b**) a+16.7 nmol/L Pb^2+^ reaction solution; (**c**) a+33.3 nmol/L Pb^2+^ reaction solution; (**d**) a+83.3 nmol/L Pb^2+^ reaction solution; (**e**) a+166.7 nmol/L Pb^2+^ reaction solution; (**f**) a+333.3 nmol/L Pb^2+^ reaction solution; (**g**) a+500 nmol/L Pb^2+^ reaction solution; (**h**) a+666.7 nmol/L Pb^2+^ reaction solution.

DNAzyme catalytic strand hybridize with substrate strands to form double-stranded DNA (dsDNA). In pH 8.0 Tris-HCl buffer solution and 6.7 mmol/L NaCl, AuNP_c_ were aggregated to the AuNP_c_ aggregations which exhibited a strong RRS peak at 370 nm. Upon addition of Pb^2+^, the substrate chain of dsDNA could be cracked catalytically by Pb^2+^ to produce a short single-stranded DNA (ssDNA) that adsorbed on the AuNPc surface to form stable AuNPc -ssDNA conjugate to prevent aggregation by NaCl, With the increase of Pb^2+^ concentration, the RRS peak linear decreased at 370 nm (Fig. 2). The AuNPc-ssDNA probe of the apt-AuNP_c_-Pb^2+^ system reaction solution has strong catalytic effect on the slow reaction between H_2_O_2_ and HAuCl_4_, the products gold nanoparticles had a stronger RRS peak at 370 nm, with the increase of Pb^2+^ concentration, the RRS peak linear increased at 370 nm (Fig. 3).

### SERS spectra

**Figure 4:**
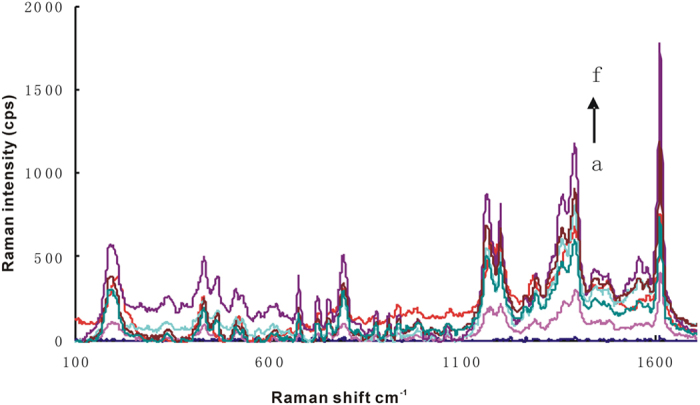
SERS spectrum of AuNP_B_-HAuCl_4_-H_2_O_2_-VBB system. (**a**) 4.48 μmol/L HAuCl_4_+0.67 mmol/L HCl+3.33 mmol/LH_2_O_2_+1.3 μmol/L VBB (**b**) a+19 ng/mL AuNP_B_; (**c**) a+38 ng/mL AuNP_B_; (**d**) a+95 ng/mL AuNP_B_; (**e**) a+190 ng/mL AuNP_B_; (**f**) a+285 ng/mL AuNP_B_.

**Figure 5:**
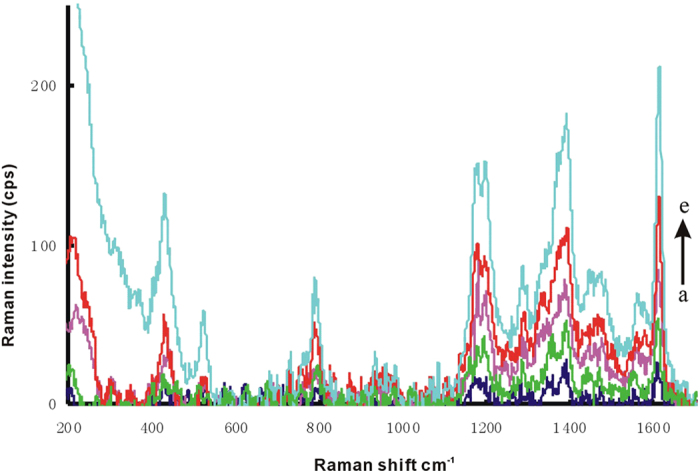
SERS spectrum of Pb^2+^ reaction solution-HAuCl_4_-H_2_O_2_-VBB nanocatalytic system. (**a**) 4.48 μmol/L HAuCl_4_+0.67 mmol/L HCl+3.33 mmol/L H_2_O_2_+1.3 μmol/L VBB; (**b**) a+50 nmol/L Pb^2+^ reaction solution; (**c**) a+167 nmol/L Pb^2+^ reaction solution; (**d**) a+250 nmol/L Pb^2+^ reaction solution; (**e**) a+500 nmol/L Pb^2+^ reaction solution.

Au^3+^ was reduced to Au and growing around the surface of nano-gold under the action of reducing agent H_2_O_2_, and the irregular shape, big particle size of nanoparticles was obtained. Upon addition of Rhs, it was adopted on the surface of gold nanoparticles which exhibited SERS peaks at 618 cm^−1^, 732 cm^−1^, 1199 cm^−1^, 1277 cm^−1^, 1356 cm^−1^, 1507 cm^−1^, 1527 cm^−1^ and 1645 cm^−1^. Among them, the SERS peak at 1645 cm^−1^ is the biggest, and the SERS peak intensity linearly increased with the concentration of AuNP_B_ increasing (Fig. S10). Upon addition of VBB, VBB molecular probes exhibited SERS peaks at 795 cm^−1^, 1167 cm^−1^, 1200 cm^−1^, 1364 cm^−1^, 1394 cm^−1^ and 1612 cm^−1^. Among them, the SERS peak at 1612 cm^−1^ is the biggest, and the SERS peak intensity linearly increased with the concentration of AuNP_B_ increasing (Fig. 4). Upon addition of Tibetan red T, Tibetan red T molecular probes exhibited SERS peaks at 349 cm^−1^, 612 cm^−1^, 1240 cm^−1^, 1372 cm^−1^, 1551 cm^−1^ and 1639 cm^−1^. Among them, the SERS peak at 1372 cm^−1^ is the biggest, and the SERS peak intensity linearly increased with the concentration of AuNP_B_ increasing (Fig. S11), and we can know that the SERS signal strength of Tibetan red T is weaker than that of RhS and VBB. When Rh6G was used as SERS probe, the SERS signal is very weak. When PdNPs solution was used as catalyst and VBB was used as SERS probe, with the increase of PdNPs concentration, the SERS peak linearly increased at 1612 cm^−1^ (Fig. S12). For the apt-nanogold-Pb^2+^ catalytic system, VBB and RhS was used as SERS probe respectively, the SERS peak intensity linearly increased with the concentration of AuNP_c_ increasing (Fig. 5, Fig. S13).

### Scanning electron microscopy(SEM)

**Figure 6:**
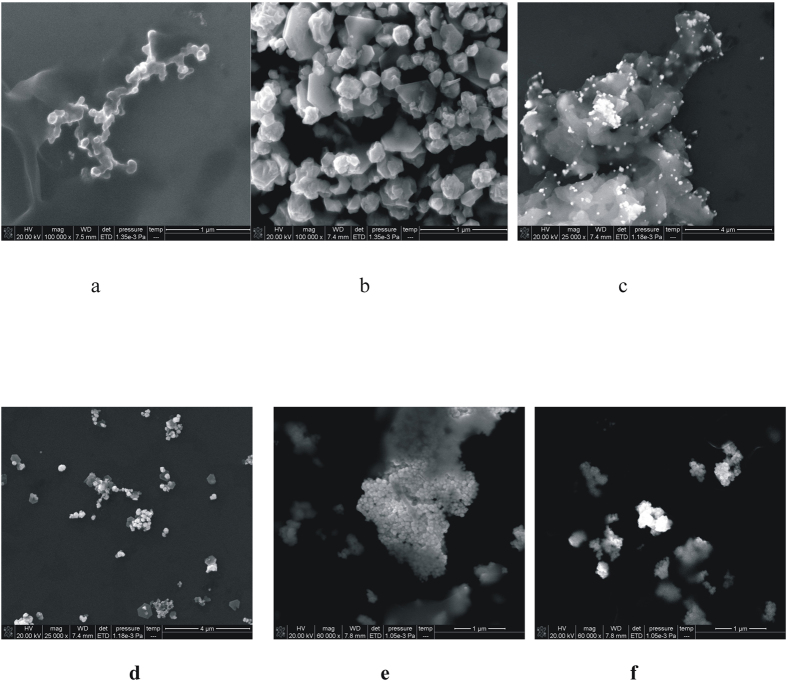
SEM of the nanoparticles. (**a**) 0.67 mmol/L HCl+4.48 μmol/L HAuCl_4_+3.33 mmol/L H_2_O_2_; (**b**) 0.67 mmol/L HCl +4.48 μmol/L HAuCl_4_+3.33 mmol/L H_2_O_2_+19 ng/mL AuNP_B_; (**c**) 0.67 mmol/L HCl +4.48 μmol/L HAuCl_4_+3.33 mmol/L H_2_O_2_+57 ng/mL AuNP_B_; (**d**) 0.67 mmol/L HCl+0.152 μg/ml AuNP_B_+3.33 mmol/L H_2_O_2_ + 19 ng/mL Apt- AuNP_B_; (**e**) 26.7 μM pH 8.0Tris-HCl-0.33 μL hybrid solution-63.7 ng/mL AuNP_C_- 0.067 mmol/L NaCl- 0.67 mmol/L HCl +4.48 μmol/L HAuCl_4_+3.33 mmol/L H_2_O_2_-16.7 nmmol/L Pb^2+^; (**f**) 26.7 μM pH 8.0Tris-HCl-0.33 μL hybrid solution -63.7 ng/mL AuNP_C_-0.067 mmol/L NaCl-0.67 mmol/L HCl+4.48 μmol/L HAuCl_4_+ 3.33 mmol/L H_2_O_2_- 333 nmmol/L Pb^2+^.

According to the procedure to get the aptamer reaction solution, a 1.0 mL the solution was taken into a 1.5 mL centrifuge tube, and centrifuged in 15000 r/min for 20 min before abandoned the supernatant. A 1.0 mL water was added into the centrifuge tube and dispersed by ultrasonic 30 min, and centrifuged again. The operation was repeated, and the dispersed sample solution was dropped onto a silicon wafers and dried naturally, then the scanning electron microscope (SEM) was recorded. The size of gold nanoparticles and silver nanoparticles are uniform and small (Fig. S14a,b). Different concentration of AgNO_3_ was added to preparation of AuNR_1_, AuNR_2_, AuNR_3_, the diameter of them was 32 nm, 37 nm, 43 nm respectively (Fig. S14c–e). For AuNP_B_-HAuCl_4_-H_2_O_2_ system, in the absence of AuNP_B_, the reaction of H_2_O_2_ and HAuCl_4_ is slow under the condition of 0.67 mmol/L HCl medium and 60 °C, and the products of gold nanoparticles is less (Fig. 6a). Upon addition of the AuNP_B_, the reaction was accelerated by nano-catalyst of small gold nanoparticles (AuNP_B_), and it would reacted to form a large number of gold nanoparticles which was irregular shape, big particle size. With the increase of AuNP_B_ concentration, the amount of gold nanoparticles increased which had high SERS and RRS singals (Fig. 6b,c). When AuNPc-ssDNA was used as catalyst, the products gold nanoparticles were gathered into small clusters (Fig. 6d). Compared with the same concentration of nangold, the catalytic activity of nanogold modified by aptamer was better due to its size was smaller. For the apt-AuNPc-Pb^2+^ system, with the increase of Pb^2+^ concentration, the amount of reaction product gold nanoparticles increased (Fig. 6e,f).

### Research of gold nanoparticle-HAuCl_4_-H_2_O_2_ reaction

The effect of HCl concentration was examined. It was found that the influence of hydrochloric acid concentration has a great influence on the formation of gold nanoparticles. The results showed that the Δ*I* value reached its maximum when the concentration was 0.5 mmol/L (Fig. S15). But under this condition, the color of the blank was pink and the RRS value was 3506 which indicated that the blank had formed a large number of gold nanoparticles. Thus, the 0.67 mmol/L was chosen for use which RRS value was 506 and colorless. The effect of HAuCl_4_ and H_2_O_2_ concentration was studied. The results showed that the Δ*I* value reached its maximum when the concentration was 4.48 μmol/L and 3.33 mmol/L H_2_O_2_ respectively (Figs S16 and S17). Thus a 4.48 μmol/L of HAuCl_4_ and 3.33 mmol/L H_2_O_2_ solutions were chosen for use. The effect of the incubation temperature was considered, when the temperature was greater than 60 °C, with the increase of temperature, the RRS value and color of blank increased gradually. When the incubation temperature was 60 °C, the blank RRS value was 745 and the color was colorless, meanwhile the catalytic reaction was very slow below 60 °C. Therefore the best temperature was 60 °C (Fig. S18). The effect of incubation time on the catalytic reaction was considered, a fixed reaction time of 15 min was chosen for use, giving a good compromise between high sensitivity, short analytical time and low blank (Fig. S19). After quenching the catalytic reaction, cooling with ice water to quench the reaction, the scattering intensity was constant within 90 min (Fig. S20). The effect of Raman probe RhS, VBB and Tibetan red T concentration were examined, and the results showed that the Δ*I* value reached its maximum when their concentration were 7 μmol/L, 13.2 μmol/L, 6.7 μmol/L respectively (Figs S21–S23).

**Figure 7:**
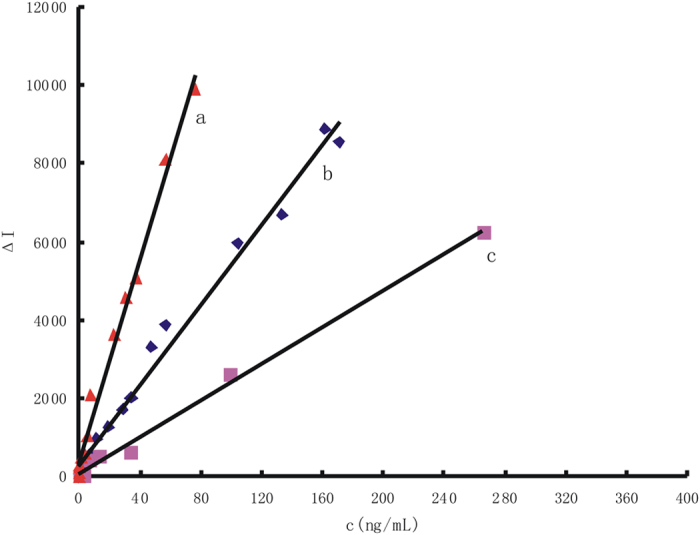
Relationship between nanogold concentration of H_2_O_2_-HAuCl_4_ catalytic system with I_RRS_. (**a**) 4.48 μmol/L HAuCl_4_-0.67 mmol/L HCl-3.33 mmol/L H_2_O_2_-AuNP_B_; (**b**) 4.48 μmol/L HAuCl_4_-0.67 mmol/L HCl-3.33 mmol/L H_2_O_2_-AuNP_c_; (**c**) 4.48 μmol/L HAuCl_4_-0.67 mmol/L HCl-3.33 mmol/L H_2_O_2_-AuNPs.

**Figure 8:**
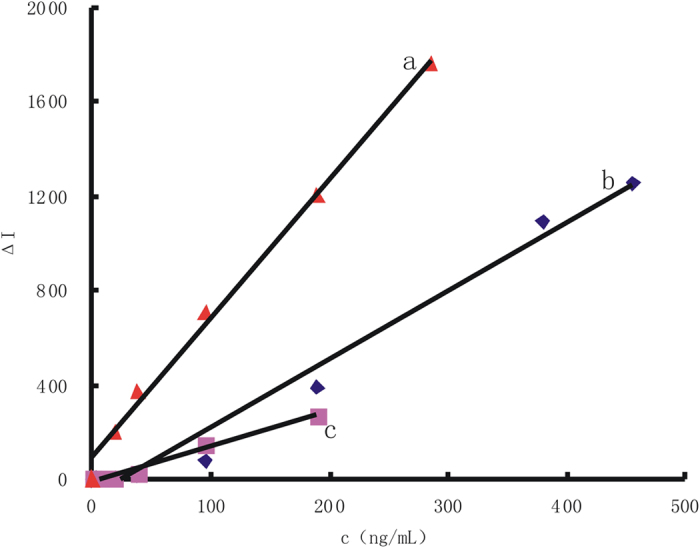
Relationship between NG concentration of H_2_O_2_-HAuCl_4_ catalytic system with I_SERS._ (**a**) 4.48 μmol/L HAuCl_4_-0.67 mmol/L HCl-3.33 mmol/L H_2_O_2_-AuNP_B_-1.3 μmol/L VBB; (**b**) 4.48 μmol/L HAuCl_4_-0.67 mmol/L HCl-3.33 mmol/L H_2_O_2_-AuNP_B_-6.97 μmol/L RhS; (**c**) 4.48 μmol/L HAuCl_4_-0.67 mmol/L HCl-3.33 mmol/L H_2_O_2_-AuNP_B_-6.7 mmol/LTibetan red T.

The gold nanoparticle reaction of HAuCl_4_-H_2_O_2_ was slow in diluted HCl solution at 60 °C and was accelerated by nano-catalyst of small gold nanoparticles (AuNP_B_, AuNP_c_, AgNPs, PdNPs and PtNPs). Under the optimal conditions, the RRS intensity for different AuNP concentrations (C) was recorded and the working curves were drawn according the relationship between C and their corresponding Δ*I* values. We have investigated the influence of different kinds of AuNP on the working curve (Fig. 7, Fig. S24). Table 1 showed that the AuNP_B_ system was the best, with the most wide linear range and lowest detection limit. We have investigated the influence of different size of AuNR on the working curve (Fig. S25), Table 1 showed that with the increase of AuNR particle size, the catalytic activity was weaker. As for AuNP_B_-HAuCl_4_-H_2_O_2_ system, under the optimal conditions, RhS, VBB, and Tibetan red T was added as SERS probe respectively, the increased SERS intensity responded linearly with the concentration of AuNP_B_ over 3.8–456, 19–285, 4–190 ng/mL respectively, with a linear regression equation of Δ*I*_*1645 cm−*1_ = 2.28 *C* + 72.77, Δ*I*_*1612 cm−*1_ = 5.94 *C* + 86, Δ*I*_*1372 cm−*1_ = 1.47 *C*−9.1 respectively (Fig. 8). Results showed that when VBB used as SERS probe was the most sensitive. When PdNPs was used as catalyst and VBB was used as SERS probe, the increased SERS intensity at 1612 cm^−1^ responded linearly with the concentration of PdNPs over 500–5950 ng/mL, with a linear regression equation of Δ*I*_*1612 cm−*1_ = 14.71 *C* + 2.06 (Fig. S26). We have investigated the influence of different AuNP on the working curve, and the results (Table 1) showed that the AuNP_B_ system was the best, with the lowest detection limit.

The gold nanoparticle reaction of HAuCl_4_-H_2_O_2_ was slow in diluted HCl solution at 60 °C. Upon addition of the nanoparticles, HAuCl_4_ would be adsorbed on the surface of nanoparticles catalyst. The surface energy was higher when the nanoparticles particle size was very small, and it can absorb a large number of HAuCl_4_ in the surface of the nanoparticles. When Au^3+^ was reduced to Au and growing around the nano-gold surface under the action of reducing agent H_2_O_2_, irregular shape, big particle size of nanoparticles were obtained. The products had a strong RRS signal because of the particle size was very large. When VBB, RhS and Tibetan red T were added as SERS probe respectively, the products had strong SERS signal because of the shape of gold nanoparticles was random. From Table 1, we can know that the AuNP_B_ RRS system was the best.

### Optimization of aptamer detection of Pb^2+^ system analysis conditions

The effect of Tris-HCl concentration and pH were examined, the results showed that the Δ*I* value reached its maximum when the concentration was 4 mmol/L and pH was 8.0 (Figs S27 and 28). The effect of AuNP_c_ and NaCl concentration were examined, the results showed that the Δ*I* value reached its maximum when the concentration were 9.55 μg/mL and 10 mmol/L respectively (Figs S29 and 30), thus, 9.55 μg/mL of AuNP_c_ and 10 mmol/L of NaCl solution were chosen for use.

### Effect of foreign substances

According to the procedure, the effect of foreign substances on the determination of 0.167 μmol/L Pb^2+^ was tested, with a relative error within ±10%. Results (Table S1) showed that common ions did not interfere with the determination, which indicated that this method had good selectivity.

### Working Curve

Under the optimal conditions, the RRS intensity for different Pb^2+^ concentrations (C) were recorded and the working curves were drawn according the relationship between C and their corresponding Δ*I* values. With the increase of Pb^2+^ concentration, the RRS peak increased at 370 nm and the decreased RRS intensity responded linearly with the concentration of Pb^2+^ over 125–425 nmol/L with a linear regression equation of Δ*I*_*370 nm*_ = 1.26C−20.56, coefficient R^2^ of 0.9836. For the apt-nanogold-Pb^2+^ catalytic system, The increased RRS intensity at 370 nm responded linearly with the concentration of Pb^2+^ over 16.7 – 666.7 nmol/L, the linear regression equation is Δ*I*_*370 nm*_ = 9.85 C + 470, coefficient R^2^ of 0.9856 (Fig. S31). VBB and RhS were added as SERS probe, the SERS intensity *I*_*1612 cm−*1_ and *I*_*1645 cm−*1_ responded linearly with the concentration of Pb^2+^ over 17–250, 17–167 nmol/L respectively (Fig. S32).

### Sample analysis

Three natural water samples were filtered to obtain water sample solutions, and were analyzed according to the procedures. Results (Table S2) showed that two of them had been detected out of Pb^2+^. A known amount of Pb^2+^ was added into the water sample to obtain the recovery. The relative standard deviation was in the range of 4.4–5.5%, and the recovery was in the range of 98.0–102%.

## Discussion

### Analytical principle

**Figure 9:**
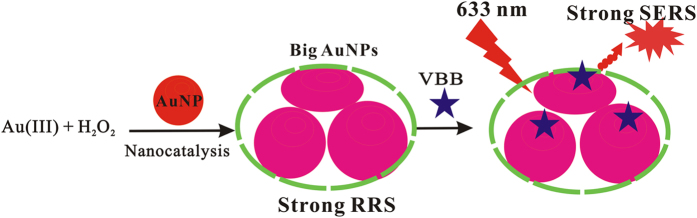
Small AuNPs catalyze the H_2_O_2_ reduction of HAuCl_4_ to big AuNPs with SERS and RRS effects.

Nanocatalytic reaction is an important way to amplify the signal of analysis method, so explore a new method to use nanocatalytic reaction is great significance. It was found that, the gold nanoparticle reaction of HAuCl_4_-H_2_O_2_ is slow in diluted HCl solution at 60 °C, upon addition of nanoparticles such as AuNP_B_, AuNP_c_, AuNR, AgNPs, PdNPs and PtNPs, HAuCl_4_ would be adsorbed on the surface of nanoparticles catalyst. The specific surface area is larger because of the nanoparticles particle size is very small, therefore it can absorb a large number of HAuCl_4_ in the surface of the nanoparticles, owing to the fact that the small nanoparticles have a high surface energy. When Au^3+^ was reduced to Au and growing around the nano-gold surface under the action of reducing agent H_2_O_2_, it can obtain irregular shape, big particle size of nanoparticles (Fig. 9), which have highly SERS signals and RRS signals. Thus the nanogold catalytic reaction can be used to build SPR-S analysis platform.

**Figure 10:**
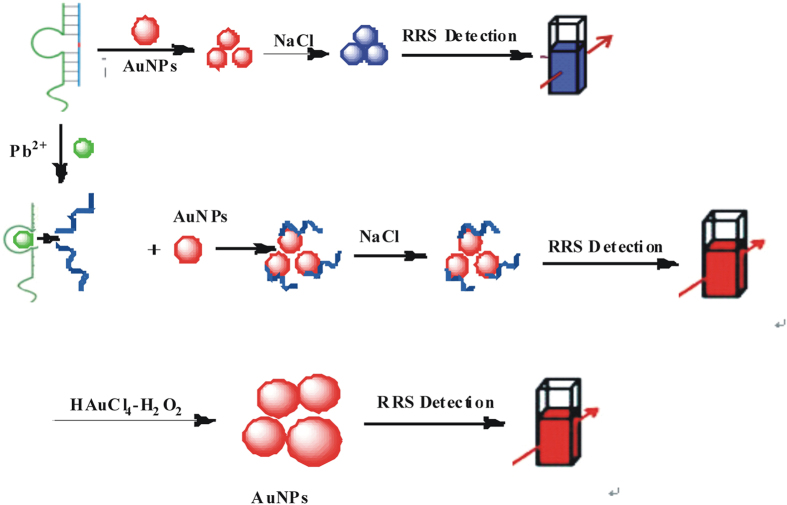
Principle of aptamer nanogold catalytic detection of Pb^2+^ with RRS effects.

The DNAzyme catalytic strand hybridized with substrate strands to form double-stranded DNA (dsDNA) which couldn’t protect AuNP_c_ in pH 8.0 Tris-HCl buffer solution containing 6.7 mmol/L NaCl, and were aggregated to AuNP_c_ aggregations with a strong RRS peak at 370 nm. Upon addition of Pb^2+^, the substrate chain of dsDNA could be cracked catalytically by Pb^2+^ to produce a short single-stranded DNA (ssDNA) that adsorbed on the AuNPc surface to form stable AuNPc-ssDNA conjugate to prevent aggregation by NaCl. Combining the nanocatalytic SPR-RRS analytical platform and the DNAzyme cracking reaction, the AuNPc-ssDNA conjugates have strong catalytic activity to HAuCl_4_-H_2_O_2_ particles reaction, and its product gold nanoparticles had a stronger RRS peak at 370 nm. With the increase of Pb^2+^ concentration, the concentration of AuNPc–ssDNA probe increase and lead to the catalytic activity stronger (Fig. 10). Based on this, the new sensitive RRS and SERS quantitative analysis methods were developed for detection of Pb^2+^.

## Conclusion

In 0.67 mmol/L HCl medium at 60 °C, HAuCl_4_ adsorbed on the surface of nanoparticles catalyst, Au^3+^ was reduce to Au and growing around the nano-gold surface, the products have highly SERS signals and RRS signal, thus the AuNP-HAuCl_4_-H_2_O_2_ nanogold catalytic reaction RSS and SERS analysis platform were built. The AuNPc-ssDNA probe of the apt-AuNP_c_-Pb^2+^ system reaction solution has strong catalytic effect on the slow reaction between H_2_O_2_ and HAuCl_4_. Combing the nanocatalysis and the DNAmyze reaction, a new nanocatalysis analysis platform was developed for the detection of Pb^2+^ by the RRS and SERS, with advantages of high sensitivity, good selectivity, simplicity and rapidity. Compared with the reported methods, the methods are easier to operate and more sensitive. Further more, it is a rapid RRS and SERS quantitative method for Pb^2+^ (Table S3).

## Methods

### Apparatus

A model of DXR smart Raman spectrometer (Thermo companies in the United States) with a laser wavelength of 633 nm and power of 2.5 mW, a model of the F-7000 Hitachi Fluorescence spectrometer (Hitachi Company, Japan), a model of the TU-1901 double-beam UV-Vis spectrophotometer (Beijing Purkinje General Instrument Co., Ltd., China), a model of FEI 200 FEG field emission scanning electron microscope (Dutch philips), and a model of C-MAG HS7 incubation magnetic stirrer (Germany IKA company) were used.

### Reagents

A 1.0 μmol/L DNAzyme catalytic strand with sequence of 5′-(T)10 CAT CTC TTC TCC GAG CCG GTC GAA ATA GTG AGT-3′, 1.0% HAuCl_4_, 1.0% sodium citrate, 10 mmol/L sodium borohydride, 0.2 mol/L cetyltrimethyl ammonium bromide (CTAB), 4.0 mmol/L AgNO_3_, 77.8 mmol/L vitamin C (VC), 0.01 mol/L HCl, 0.3% H_2_O_2_ (0.1 mol/L), 50 mmol/L pH 7.4 Tris-HCl, 50 mmol/L pH 8.0 Tris-HCl, 5 × 10^−5 ^mol/L PdCl_2_ and 1.45 × 10^−2 ^mol/L PdCl_2_, 2.9 × 10^−2 ^mol/L HPtCl_6_ and 5.23 × 10^−5^ mol/L RhS solution were prepared. A pH 7.0 Na_2_HPO_4_-citric acid buffer solution was prepared as follows, a 16.5 mL 0.2 mol/L Na_2_HPO_4_ and 3.5 mL 0.1 mol/L citric acid solution were mixed together to obtain a concentrations 0.16 mol/L Na_2_HPO_4_. A 1.0 × 10^−3 ^mol/L VBB solution was prepared as follows, 0.0250 g VBB was dissolved in 5.0 mL ethanol, and diluted to 50 mL with water. The nanosols and ssDNA-AuNP were prepared as in the SI^37^.

### Procedure of HAuCl_4_- nanoparticles -H_2_O_2_ system

A 80 μL 0.1% HAuCl_4_ (84 μmol/L), 100 μL 0.01 mol/L HCl, a certain amount of nanoparticles including AuNP_B_, AuNP_c_, AuNR, AgNPs, PdNPs and PtNPs, and 50 μL 0.3% (0.1 mol/L) H_2_O_2_ were added into a 5 mL marked test tube and mixed well, and diluted to 1.5 mL. The mixture was heated at 60 °C for 15 min, cooling with ice water to quench the reaction. A part of the solution was transferred into a 1 cm quartz cell. The RRS spectra were recorded by synchronous scanning excited wavelength *λ*_ex_ and emission wavelength *λ*_em_ (*λ*_ex_−*λ*_em_ = Δ*λ* = 0), a PMT voltage of 400 v, both excited and emission slit width of 5 nm, emission filter of 1%T attenuator on fluorescence spectrophotometer. The RRS intensity at 370 nm (*I*_370 nm_) and the blank value (*I*_370 nm_)_0_ without nanoparticles were recorded. The value of Δ*I*_370 nm_ = *I*_370 nm_−(*I*_370 nm_)_0_ was calculated. 200 μL 1.0 × 10^−5 ^mol/L VBB, 20 μL 5.23 × 10^−5^ mol/L RhS or 100 μL 1 × 10^−4^ mol/L tibetan red T was added in the mixture respectively, The SERS intensity corresponding at 1612 cm^−1^, 1645 cm^−1^, 1370 cm^−1^ and the blank value *I*_*0*_without nanoparticles were recorded. The value of Δ*I* = *I* − *I*_*0*_ was obtained.

### Procedure of apt-AuNP_c_-Pb^2+^ system

A 500 μL 2 μmol/L Substrate strand, 500 μL 1 μmol/L DNAzyme catalytic strand, 1 mL 50 mM pH 7.4 Tris-Hcl buffer solution and 50 μL 1 mol/L NaCl were mixed well, incubated at 65 °C water bath for 10 min, then gradually cooled to room temperature over 2 h, and hybrid solution was obtained. In a 5 mL marked test tube 120 μL 50 mM pH 8.0 Tris-Hcl buffer solution, 50 μL hybrid solution, a certain amount of Pb^2+^ was added respectively, mixed well and diluted to 1.5 mL. Then the tube was placed at 37 °C water bath for reaction 60 min before cooling with ice water to quench the reaction. After that 250 μL AuNP_c_ and 30 μL 0.5 mol/L NaCl were added in the mixture and mixed well to obtain Pb^2+^ aptamer reaction solution, then a part of the solution was transferred into a 1 cm quartz cell. The RRS spectra were recorded by synchronous scanning excited wavelength *λ*_ex_ and emission wavelength *λ*_em_ (*λ*_ex_−*λ*_em_ = Δ*λ* = 0), a PMT voltage of 450 v, both excited and emission slit width of 5 nm, The RRS intensity at 370 nm (*I*_370 nm_) and the blank value (*I*_370 nm_)_0_ without Pb^2+^ were recorded. The value of Δ*I*_370 nm_ = (*I*_370 nm_)_0_ − *I*_370 nm_ was calculated.

### Procedure of apt-nanogold-Pb^2+^ catalytic system

A 80 μL 0.1% HAuCl_4_ (84 μmol/L),100 μL 0.01 mol/L HCl, 100 μL Pb^2+^ aptamer reaction solution and 50 μL 0.3% (0.1 mol/L) H_2_O_2_ were added into a 5 mL marked test tube and mixed well, and diluted to 1.5 mL. The mixture was heated at 60 °C for 15 min, cooling with ice water to quench the reaction. A part of the solution was transferred into a 1 cm quartz cell. The RRS spectra were recorded by synchronous scanning excited wavelength *λ*_ex_ and emission wavelength *λ*_em_ (*λ*_ex_ − *λ*_em_ = Δ*λ* = 0), a PMT voltage of 400 v, both excited and emission slit width of 5 nm, emission filter of 1%T attenuator on fluorescence spectrophotometer. The RRS intensity at 370 nm (*I*_370 nm_) and the blank value (*I*_370 nm_)_0_ without Pb^2+^ were recorded. The value of Δ*I*_370 nm_ = *I*_370 nm_ − (*I*_370 nm_)_0_ was calculated. 200 μL 1.0 × 10^−5^ mol/L VBB 20 μL or 5.23 × 10^−5 ^mol/L RhS was added in the mixture, The SERS intensity at 1612 cm^−1^ and the blank value *I*_*0*_without Pb^2+^ were recorded. The value of Δ*I* = *I* – *I*_*0*_was obtained.

## Additional Information

**How to cite this article**: Ye, L. *et al*. A novel and highly sensitive nanocatalytic surface plasmon resonance-scattering analytical platform for detection of trace Pb ions. *Sci. Rep*. **6**, 24150; doi: 10.1038/srep24150 (2016).

## Supplementary Material

Supplementary Information

## Figures and Tables

**Table 1:** Different nanoparticles catalytic systems analysis feature.

system	Detection method		The regression equation	Linear range (ng/mL NP)	The correlation coefficient
H_2_O_2_-HAuCl_4_-AuNP_B_	RRS		Δ*I* = 131 C + 300	0.038–76	0.9951
	Different	RhS	Δ*I* = 5.9 C + 86	3.8–456	0.9898
	probe	VBB	Δ*I* = 2.9 C−73	19–285	0.9966
	SERS	Tibetan red T	Δ*I* = 1.5 C−9.1	4–190	0.9923
	UV		Δ*A* = 2.6 × 10^−3^ C+0.0406	9.5–180	0.9825
H_2_O_2_-HAuCl_4_-AuNP_c_	RRS		Δ*I* = 51C + 267	0.38–171	0.9941
	UV		Δ*A* = 2 × 10^−3^ C−0.0407	38–228	0.9808
H_2_O_2_-HAuCl_4_-AuNR_1_	RRS		Δ*I* = 0.51 C + 54	32.5–975	0.9840
H_2_O_2_-HAuCl_4_-AuNR_2_	RRS		Δ*I* = 0.37 C + 21	32.5–2600	0.9901
H_2_O_2_-HAuCl_4_-AuNR_3_	RRS		Δ*I* = 0.24 C + 73	32.5–1950	0.9784
H_2_O_2_-HAuCl_4_-AgNPs	RRS		Δ*I* = 23 C + 73	3.3–265	0.9971
	UV		Δ*A* = 0.001 C + 0.0203	13–265	0.9701
H_2_O_2_-AuCl_4_-PdNPs	RRS		Δ*I* = 0.12 *C* + 2.6	200–9920	0.9939
	SERS		Δ*I* = 14.71 *C* + 2.06	500–5950	0.9913
	UV		Δ*A* = 2 × 10^−5^*C* + 0.011	298–1587	0.9939
H_2_O_2_-HAuCl_4_-PtNPs	RRS		Δ*I* = 0.05 *C* + 21	200–600	0.9729
H_2_O_2_-HAuCl_4_-AuNP_c_-Apt	RRS		Δ*I* = 70.7 C + 257	0.95–76	0.9936
