# Supplementary material for: A novel and highly sensitive nanocatalytic surface plasmon resonance-scattering analytical platform for detection of trace Pb ions
Source: Sci Rep. 2016 Apr 13;6:24150. doi: 10.1038/srep24150 (PMC4829859; doi:10.1038/srep24150)
Supplement: Supplementary Information [file srep24150-s1.doc]

**A novel and highly sensitive nanocatalytic surface plasmon resonance-scattering analytical platform for detection of trace Pb ions**

Lingling Ye^§^, Guiqing Wen^§^, Huixiang Ouyang, Qingye Liu, Aihui Liang^*^ Zhiliang Jiang^*^

Key Laboratory of Ecology of Rare and Endangered Species and Environmental Protection of Ministry Education, Guangxi Normal University, Guilin 541004, China

**Keyword:** DNAzyme, Pb(II), nanogold, nanocatalytic amplification, SPR-S

**1. Preparation of nanoparticles**

**1.1 Preparation of gold nanoparticles**

**Sodium citrate reduction method (AuNP_c_).** Into a 50 mL boiling water with stirring, a 0.5 mL 1% HAuCl_4_ and 3.5mL 1% trisodium citrate were added rapidly successively. After boiling for 10 min, the color became from colorless to wine red. The mixture was continued stirring to room temperature, and then diluted to 50 mL to obtain a concentration of 58.0 μg/mL Au nanogold in size of about 10 nm.

**NaBH_4_ reduction method (AuNP_B_).** Gold nanoparticles preparation steps are as follows ^[37]^： A 40 mL water was added into a flask, a 3.5mL 1.0% trisodium citrate and 0.500mL 1.0% HAuCl_4_were added into successively. Then, 4.0mL 0.5mg/mL NaBH_4_ was dripped slowly. After 10 min, it was diluted to 50 mL to obtain a 58 μg/mL Au nanogold sol that stored at 4 ℃.

**Preparation of different particle size gold nanorods (AuNR).** Obtaining of the golden seed solution: 10 mL 0.5 mmol/L HAuCl_4_ and 10 mL 200mmol/L CTAB were added into a flask and mixed well. The color of solution turned into bright yellow. Then 1.2mL 10mmol/L NaBH_4_ was added into successively. The color of solution turned from bright yellow to tan and keep vigorous stirring for 2 minutes before standing for 2 hours.The experimental process was carry at room temperature. Obtaining of growth solution: A 5mL 1 mmol/L HAuCl_4_ was added into three 100mL flask. And 25μL, 50μL, 75μL 4mmol/L AgNO_3_ were added into the three flasks successively. Then 5mL 200mmol/L CTAB was added, and the color changed to tan. Next 70μL 77.8mmol/L ascorbic acid was added, and the color changed immediately to colorless. Then 20μL golden seed solution was added and the color was gradually becoming deep in a few minutes, standing for 12 h. With the increasing amount of silver nitrate solution the solution color changed from deep blue to pale blue. Noted as AuNR_1_, AuNR_2_, AuNR_3_ respectively. The experimental process was carry at 27℃-30℃. The gold nanorods were centrifuged for 15 min (150 x 100 r/min) to remove residual impurities such as CTAB, then took the precipitation in the bottom and diluted with deionized water to the original volume before ultrasonic treatment for 5 min. Repeat the centrifugal and ultrasonic processing respectively to obtain a 97.5 μg/mL different particle size gold nanorods.

**1.2 Preparation of silver nanoparticles（AgNPs）**

Silver nanoparticle was synthesized through NaBH_4_ reduction process. A 40 mL water was added into a flask, a 3.5mL10g/L trisodium citrate and 385 μL 2.4×10^-2^ mol/L AgNO_3_ were added into successively. Then, 4.0mL 0.5mg/mL NaBH_4_ was dripped slowly. After 20 min, it was diluted to 50 mL to obtain a 19.9μg/mL nanosilver that stored at 4 ℃.

**1.3 Preparation of palladium nanoparticles (PdNPs)**

Palladium nanoparticle was synthesized through NaBH_4_ reduction process. A 40 mL water was added into a flask, a 3.5mL10g/L trisodium citrate and 950.0 μL 1.45×10^-2^ mol/L PdCl_2_ were added into successively. Then, 4.0mL 0.5mg/mL NaBH_4_ was dripped slowly. After 20 min, it was diluted to 50 mL to obtain a 29.75 μg/mL nanopalladium that stored at 4 ℃.

**1.4 Preparation of platinum nanoparticles (PtNPs)**

Platinum nanoparticle was synthesized through NaBH_4_ reduction process. A 40 mL water was added into a flask, a 3.5mL10g/L trisodium citrate and 200.0 μL 2.9×10^-2^ mol/L HPtCl_6_ were added into successively. Then, 4.0mL 0.5mg/mL NaBH_4_ was dripped slowly. After 20 min, it was diluted to 50 mL to obtain a 22.97 μg/mL nanoplatinum that stored at 4 ℃.

A	B	C

D

**Figure S1 RRS spectrum of AuNR**

(A)AuNR_1_; (B)AuNR_2_; (C)AuNR_3_; D: Different concentrations of AuNR linear diagram.

a: 6.5μg/mL AuNR; b: 13μg/mL AuNR; c: 26μg/mL AuNR; d: 39μg/mL AuNR.

**Figure S2 RRS spectrum of AuNP_B_-HAuCl_4_-H_2_O_2_ system**

（a）4.48µmol/L HAuCl_4_+0.67mmol/L HCl+3.33mmol/L H_2_O_2_ （b）a+0.038ng/mL AuNP_B_; （c）a+0.095ng/mL AuNP_B_； （d）a+1.9ng/mL AuNP_B_； （e）a+5.7 ng/mL AuNP_B_; （f）a+7.6 ng/mL AuNP_B_；（g）a+22.8 ng/mL AuNP_B_;（h）a+38 ng/mL AuNP_B_;（i）a+76 ng/mL AuNP_B_.

**Figure S3 RRS spectrum of AuNR_1_-HAuCl_4_-H_2_O_2_ system**

（a）4.48µmol/L HAuCl_4_+0.67mmol/L HCl+3.33mmol/L H_2_O_2_ （b）a+32.5ng/mL AuNR_1_; （c）a+65ng/mL AuNR_1_； （d）a+325ng/mL AuNR_1_； （e）a+650ng/mL AuNR_1_; （f）a+975 ng/mL AuNR_1_.

**Figure S4 RRS spectrum of AuNR_2_-HAuCl_4_-H_2_O_2_ system**

（a）4.48µmol/L HAuCl_4_+0.67mmol/L HCl+3.33mmol/L H_2_O_2_ （b）a+32.5ng/mL AuNR_2_; （c）a+162.5ng/mL AuNR_2_； （d）a+650ng/mL AuNR_2_; （e）a+1300 ng/mL AuNR_2_; （f）a+2600 ng/mL AuNR_2_.

**Figure S5 RRS spectrum of AuNR_3_-HAuCl_4_-H_2_O_2_ system**

（a）4.48µmol/L HAuCl_4_+0.67mmol/L HCl+3.33mmol/L H_2_O_2_ （b）a+32.5ng/mL AuNR_3_; （c）a+162.5ng/mL AuNR_3_； （d）a+487.5ng/mL AuNR_3_; （e）a+650 ng/mL AuNR_3_; （f）a+1300 ng/mL AuNR_33_; （g）a+1950 ng/mL AuNR_3_.

**Figure S6 RRS spectrum of PdNPs-HAuCl_4_-H_2_O_2_ system**

（a）4.48µmol/L HAuCl_4_+0.67mmol/L HCl+3.33mmol/LH_2_O_2_ （b）a+0.20µg/mLPdNPs; （c）a+0.99µg/mLPdNPs； （d）a+1.98µg/mLPdNPs； （e）a+4.96µg/mLPdNPs; （f）a+7.93µg/mLPdNPs；（g）a+9.92µg/mLPdNPs.

**Figure S7 RRS spectrum of HAuCl_4_-AuNP_c_-H_2_O_2_ system**

（a）0.67mmol/L HCl+0.152µg/mlAuNP_C_+3.33mmol/L H_2_O_2_； （b）a+0.56µmol/L HAuCl_4_； （c）a+1.12µmol/L HAuCl_4_ ； （d）a+2.24µmol/L HAuCl_4_； （e）a+2.8µmol/L HAuCl_4_； （f）a+3.36µmol/L HAuCl_4_； （g）a+4.48µmol/L HAuCl_4._

**Figure S8 RRS spectrum of H_2_O_2_-HAuCl_4_-AuNP_c_ system**

（a）4.48µmol/L HAuCl_4_+0.67mmol/L HCl+0.152µg/ml AuNP_C_ （b）a+0.33mmol/L H_2_O_2_； （c）a+0.5 mmol/L H_2_O_2_； （d）a+0.67 mmol/L H_2_O_2_； （e）a+2 mmol/L H_2_O_2_ ;（f）a+3.33 mmol/L H_2_O_2._

**Figure S9 RRS spectrum of（ssDNA-AuNP_c_）-HAuCl_4_-H_2_O_2_ system**

（a）4.48µmol/L HAuCl_4_+0.67mmol/L HCl+3.33mmol/L H_2_O_2_ （b）a+0.95ng/mL ssDNA -AuNP_c_; （c）a+7.6 ng/mL ssDNA -AuNP_c_； （d）a+28.5 ng/mL ssDNA -AuNP_c_； （e）a+38 ng/mL ssDNA -AuNP_c_; （f）a+76 ng/mL ssDNA -AuNP_c_.

**Figure S10 SERS spectrum of AuNP_B_-HAuCl_4_-H_2_O_2_-RhS system**

（a）4.48µmol/L HAuCl_4_+0.67mmol/L HCl+3.33mmol/L H_2_O_2_+0.70µmol/L RhS（b）a+3.8ng/mL AuNP_B_; （c）a+19ng/mL AuNP_B_； （d）a+38ng/mL AuNP_B_； （e）a+95ng/mL AuNP_B_; （f）a+190 ng/mL AuNP_B_；（g）a+380 ng/mL AuNP_B_;（h）a+456 ng/mL AuNP_B_.

**Figure S11 SERS spectrum of AuNP_B_-HAuCl_4_-H_2_O_2_-Tibetan red T system**

（a）4.48µmol/L HAuCl_4_+0.67mmol/L HCl+3.33mmol/L H_2_O_2_ +6.7mmol/LTibetan red T（b）a+3.8ng/mL AuNP_B_; （c）a+19ng/mL AuNP_B_； （d）a+38ng/mL AuNP_B_； （e）a+95ng/mL AuNP_B_; （f）a+190 ng/mL AuNP_B_；（g）a+295 ng/mL AuNP_B_;（h）a+380 ng/mL AuNP_B_.

**Figure S12 SERS spectrum of PdNPs-HAuCl_4_-H_2_O_2_-VBB system**

（a）4.48µmol/L HAuCl_4_+0.67mmol/L HCl+3.33mmol/L H_2_O_2_ +1.3µmol/L VBB（b）a+0.50µg/mL PdNPs; （c）a+0.99µg/mL PdNPs； （d）a+2.98µg/mL PdNPs； （e）a+3.97µg/mL PdNPs; （f）a+4.96µg/mL PdNPs；（g）a+5.95µg/mL PdNPs.

**Figure S13 SERS spectrum of Pb^2+^ reaction solution-HAuCl_4_-H_2_O_2_-RhS system**

（a）4.48µmol/L HAuCl_4_+0.67mmol/L HCl+3.33mmol/L H_2_O_2_+0.70µmol/L RhS ；（b）a+50nmol/L Pb^2+^ reaction solution；（c）a+167nmol/L Pb^2+^ reaction solution；（d）a+250nmol/L Pb^2+^ reaction solution；（e）a+500nmol/L Pb ^2+^ reaction solution.

a	b	c

d	e

**Figure S14 SEM**

(a)AuNP_B_；（b）AgNPs；（c）AuNR_1_； （d）AuNR_1_；（e）AuNR_1_.

**Figure S15 Effect of HCl concentration**

2.24µmol/L HAuCl_4_- HCl-3.33mmol/L H_2_O_2_ -152ng/mL AuNP_B_.

**Figure S16 Effect of HAuCl_4_ concentration**

HAuCl_4_- 0.67mmol/L HCl-3.33mmol/L H_2_O_2_ -152ng/mL AuNP_B_.

**Figure S17 Effect of H_2_O_2_ concentration**

4.48µmol/L HAuCl_4_- 0.67mmol/L HCl-H_2_O_2_ -152ng/mL AuNP_B_.

**Figure S18 Effect of reation temperature**

4.48µmol/L HAuCl_4_- 0.67mmol/L HCl-3.33mmol/L H_2_O_2_ -152ng/mL AuNP_B_.

**Figure S19 Effect of reation time**

4.48µmol/L HAuCl_4_- 0.67mmol/L HCl-3.33mmol/L H_2_O_2_ -152ng/mL AuNP_B_.

**Figure S20 Effect of standing time after reation**

4.48µmol/L HAuCl_4_- 0.67mmol/L HCl-3.33mmol/L H_2_O_2_ -38ng/mL AuNP_B_.

**Figure S21 Effect of the Raman probe RhS concentration**

4.48µmol/L HAuCl_4_- 0.67mmol/L HCl-3.33mmol/L H_2_O_2_ -190ng/mL AuNP_B_-RhS.

**Figure S22 Effect of the Raman probe VBB concentration**

4.48µmol/L HAuCl_4_- 0.67mmol/L HCl-3.33mmol/L H_2_O_2_ -190ng/mL AuNP_B_-VBB.

**Figure S23 Effect of the Raman probe Tibetan red T concentration**

4.48µmol/L HAuCl_4_- 0.67mmol/L HCl-3.33mmol/L H_2_O_2_ -190ng/mL AuNP_B_-Tibetan red T.

**Figure S24 The relationship between nano-particle concentration of H_2_O_2_-HAuCl_4_ catalytic system with I_RRS_**

（a）4.48µmol/L HAuCl_4_-0.67mmol/L HCl-3.33mmol/L H_2_O_2_-PdNPs；

（b）4.48µmol/L HAuCl_4_-0.67mmol/L HCl-3.33mmol/L H_2_O_2_-PtNPs.

**Figure S25 The relationship between AuNR concentration of H_2_O_2_-HAuCl_4_ catalytic system with I_RRS_**

（a）4.48µmol/L HAuCl_4_-0.67mmol/L HCl-3.33mmol/L H_2_O_2_-AuNR_1_； （b）4.48µmol/L HAuCl_4_-0.67mmol/L HCl-3.33mmol/L H_2_O_2_- AuNR_2_；（c）4.48µmol/L HAuCl_4_-0.67mmol/L HCl-3.33mmol/L H_2_O_2_-AuNR_3_.

**Figure S26 The relationship between PdNPs concentration of H_2_O_2_-HAuCl_4_ catalytic system with I_SERS_**

PdNPs-4.48µmol/L HAuCl_4_-0.67mmol/L HCl-3.33mmol/L H_2_O_2_-1.3µmol/LVBB；

**Figure S27 Effect of pH**

**4mM Tris-HCl-50μLhybrid solution -9.55µg/mL AuNP_c_-10mmol/L NaCl-0.25μmol/LPb^2+^**

Figure S28 Effect of buffer solution concentration

**pH8.0 Tris-HCl-50μLhybrid solution -9.55µg/mL AuNP_C_-10mmol/L NaCl-0.25μmol/LPb^2+^**

Figure S29 Effect of AuNP_c_ concentration

**4mmol/L-pH8.0 Tris-HCl-50μLhybrid solution -AuNP_C_-10mmol/L NaCl-0.25μmol/LPb^2+^**

Figure S30 Effect of NaCl concentration

**4mmol/L pH8.0 Tris-HCl-50μLhybrid solution -9.55µg/mL AuNP_C_- NaCl-0.25μmol/LPb^2+^**

**Figure S31 RRS working curve of ssDNA and nano-catalytic system for detection Pb^2 +^**

（a）4mM pH 8.0Tris-HCl-50μLhybrid solution -9.55µg/mLAuNP_C_-10mmol/LNaCl–Pb^2+^；

（b）4.48µmol/L HAuCl_4_+0.67mmol/L HCl+3.33mmol/L H_2_O_2_- Pb^2+^reaction solution

**Figure S32 Working curve of different SERS probe system for detection Pb^2 +^**

（a）4.48µmol/L HAuCl_4_+0.67mmol/L HCl+3.33mmol/L H_2_O_2_- Pb^2+^reaction solution-1.3µmol/LVBB;（b）4.48µmol/L HAuCl_4_+0.67mmol/L HCl+3.33mmol/L H_2_O_2_- Pb^2+^reaction solution-0.70µmol/LRhS

**Table S1 Effect of coexistent ions**

| Coexistent substance | Tolerance concentration（µmol/L） | Relative error (%) | Coexistent substance | Tolerance concentration（µmol/L） | Relative error (%) |
| --- | --- | --- | --- | --- | --- |
| Zn^2+^ | 16.7 | -2.4 | Mg^2+^ | 16.7 | 1.2 |
| Cu^2+^ | 16.7 | 0.4 | Mn^2+^ | 16.7 | -3.5 |
| Cd^2+^ | 16.7 | 7.6 | Hg^2+^ | 16.7 | 6.5 |
| Cr^3+^ | 16.7 | 8.9 | Bi^3+^ | 8.35 | 3.3 |
| Co^2+^ | 8.35 | 5.8 | Ni^2+^ | 8.35 | 5.2 |

**Table S2 Analytical results for Pb^2+^ (n=5)**

| Water | Content of Pb^2+^  (nmol/L) | Spiked Pb  (nmol/L) | Average  (nmol/L) | Recovery(%) | RSD(%) |
| --- | --- | --- | --- | --- | --- |
| Sample 1 | 4.89 | - | - | - | 5.5 |
| Sample 2 | 7.84 | - | - | - | 4.7 |
| Sample 3 | No detected | 10 | 9.80 | 98.0 | 4.5 |
|  |  | 20 | 20.4 | 102 | 4.4 |

**Table S3 Analysis features of the Pb^2+^-nanogold-DNA systems**

| Methods | Analysis principle | Analysis features | Comments | Ref. |
| --- | --- | --- | --- | --- |
| RRS( thrombin-  aptamer) | Pb^2+^ made thrombin binding aptamer and RRS enhanced | 1-120 nmol/L,DL=0.9 nmol/L | Sensitive, selective, but complex. | 25 |
| RRS ( hydride generation ) | Pb^2+^ reduced by NaBH_4_ to form PbH_4_, which reacted with Au^3+^ to produce nanogold and make RRS enhanced | 200-33600nmol/L,DL=70nmol/L | Selective, but not very sensitive. | 26 |
| SERS( DNAzyme) | DNAzyme and substrate were labled on the SERS sensor，the adding of Pb^2+^ can make the SERS decreased | 20-1000nmol/L | Selective, but complex. | 34 |
| SERS(DNAzyme -Rh6G) | DNAzyme cracked by Pb^2+^ dsDNA to produce ssDNA, which protect Au_core_Ag_shell_ from aggregating, Rh6G was used as probe | 50-700 nmol/L, DL=7nmol/L | Sensitive, but complex. | 36 |
| RRS (DNA-HAuCl_4_- H_2_O_2_) | AuNP_c_-Apt produced by DNAzyme cracking had catalytic effect on the HAuCl_4_-H_2_O_2_ reaction | 16.7-666.7 nmol/L | Selective, simple, low cost. | This paper |
| SERS(-HAuCl_4_ -H_2_O_2_) | AuNP_c_-Apt had catalytic effect on the HAuCl_4_-H_2_O_2_ reaction to form AuNP and VBB was used as SERS probe | 50-500 nmol/L | Selective, Lable-free, low cost. | This paper |

d

a

d

a

d

a

i

a

f

a

a

f

a

a

g

a

a

g

a

a

g

a

f

a

f

a

h

a

h

a

f

a

a

e

a
